# Revealing New Candidate Genes for Teat Number Relevant Traits in Duroc Pigs Using Genome-Wide Association Studies

**DOI:** 10.3390/ani11030806

**Published:** 2021-03-13

**Authors:** Yang Li, Lei Pu, Liangyu Shi, Hongding Gao, Pengfei Zhang, Lixian Wang, Fuping Zhao

**Affiliations:** 1 Key Laborary of Animal Genetics, Breeding and Reproduction (Poultry) of Ministry of Agriculture, Institute of Animal Sciences, Chinese Academy of Agricultural Sciences, Beijing 100193, China; 18331279970@163.com (Y.L.); liangyu_shi@foxmail.com (L.S.); zhangpengfei3236@163.com (P.Z.); 2 Tianjin Key Laboratory of Agricultural Animal Breeding and Healthy Breeding, College of Animal Science and Animal Medicine, Tianjin Agricultural University, Tianjin 300384, China; pulei87@126.com; 3 Center for Quantitative Genetics and Genomics, Aarhus University, 8830 Tjele, Denmark; hongding.gao@qgg.au.dk

**Keywords:** number of teats, SNP, candidate genes, Duroc pigs

## Abstract

**Simple Summary:**

Number of teats is very important for lactating sows. We conducted genome-wide association studies (GWAS) and estimated the genetic parameters for traits related to teat number. Results showed that there were nine and 22 SNPs exceeding genome-wide significance and suggestive significance levels, respectively. Eighteen genes annotated near them were concentrated on chromosomes 7 and 10. Among them, three new candidate genes were located on the genomic regions around the significant SNPs. Our findings provide new insight into investigating the complex genetic mechanism of traits related to teat number in pigs.

**Abstract:**

The number of teats is related to the nursing ability of sows. In the present study, we conducted genome-wide association studies (GWAS) for traits related to teat number in Duroc pig population. Two mixed models, one for counted and another for binary phenotypic traits, were employed to analyze seven traits: the right (RTN), left (LTN), and total (TTN) teat numbers; maximum teat number on a side (MAX); left minus right side teat number (LR); the absolute value of LR (ALR); and the presence of symmetry between left and right teat numbers (SLR). We identified 11, 1, 4, 13, and 9 significant SNPs associated with traits RTN, LTN, MAX, TTN, and SLR, respectively. One significant SNP (MARC0038565) was found to be simultaneous associated with RTN, LTN, MAX and TTN. Two annotated genes (*VRTN* and *SYNDIG*1*L*) were located in genomic region around this SNP. Three significant SNPs were shown to be associated with TTN, RTN and MAX traits. Seven significant SNPs were simultaneously detected in two traits of TTN and RTN. Other two SNPs were only identified in TTN. These 13 SNPs were clustered in the genomic region between 96.10—98.09 Mb on chromosome 7. Moreover, nine significant SNPs were shown to be significantly associated with SLR. In total, four and 22 SNPs surpassed genome-wide significance and suggestive significance levels, respectively. Among candidate genes annotated, eight genes have documented association with the teat number relevant traits. Out of them, *DPF3* genes on *Sus scrofa* chromosome (SSC) 7 and the *NRP1* gene on SSC 10 were new candidate genes identified in this study. Our findings demonstrate the genetic mechanism of teat number relevant traits and provide a reference to further improve reproductive performances in practical pig breeding programs.

## 1. Introduction

The teat number (NT) is an important determinant of the maternal ability of sows [1], which can in turn influence their reproductive efficiencies. The teat number directly affects suckling piglet survival and is a limited factor for increasing the number of weaned piglets [2]. Therefore, NT is always considered as an economically important trait that significantly influences reproductive efficiencies in pig breeding.

The teat number, unlike other reproductive traits, is an inborn trait that is less affected by environmental factors [3]. Heritabilities of TN in pig populations ranged from 0.10 to 0.70 even though most of them tend to converge on a moderate value for this trait [4,5,6,7,8,9]. Zhuang et al. reported that heritabilities of Canadian Duroc and American Duroc were 0.34 and 0.19, respectively [9]. The number of teats can vary in different pig breeds, ranging from 8 to 25. The largest total number of NT of 25 has been documented in Erhualian pig that is one of Chinese indigenous pig breeds [10]. However, the number of teats of Duroc ranges from 8 to 16 [9,11]. Breeding programs have achieved remarkable improvements in litter sizes of pigs in the last few decades. In 2009, about one-quarter of the largest litter size of purebred Danish Large White and Landrace pigs were larger than 18 [12]. The increased litter size in pigs has led to many sows incapable of nurturing all of their piglets owing to the limited. The sucking piglet competition for teats results in increased preweaning mortality because of starvation and crushing [13]. Therefore, NT should be added in breeding project to ensure that sows can raise all their piglets to increase the number of piglets per sow per year [14,15,16].

Biologically, the development of embryonic mammary glands requires the coordination of many signaling pathways to direct cell shape changes, cell movements, and cell-cell interactions that are necessary for proper morphogenesis of mammary glands [17]. To date, 602 quantitative trait loci (QTLs) have been identified as the number of teats and different between sides covering almost the whole porcine genome (https://www.animalgenome.org/cgi-bin/QTLdb/SS/ontrait?trait_ID=722, accessed on 4 March 2021). One of the most important QTLs for the number of teats *VRTN* gene is considered to be the most promising candidate gene for this QTL [18]. The causative genes or variants need further discovering.

In the current study, we analyzed the complex genetic mechanism of differences in the number and location of NT, and further provide a theoretical reference for breeding pigs. The aim of the present study was to identify SNPs and candidate genes associated with NT traits in a Duroc pig population genotyped with a dense SNP array.

## 2. Materials and Methods

### 2.1. Ethics Statement

All animals were treated following the guidelines established by the Council of China for Animal Welfare. The experimental protocols were approved by the Science Research Department of the Institute of Animal Sciences, Chinese Academy of Agricultural Sciences (CAAS) (IASCAAS-AE-07) (Beijing, China).

### 2.2. Phenotypic Data

Data on NT were obtained from a total of 982 American Duroc pigs born from 2014 to 2016 on a commercial company in Henan province. The teat counts were recorded in the right and left sides of piglets at birth. Phenotypes evaluated were the right (RTN), left (LTN), and total (TTN) teat numbers. Moreover, other four traits constructed were also analyzed, including left minus right side teat number (LR), the absolute value of LR (ALR), the presence of symmetry between left and right teat numbers (SLR), and maximum teat number on a side (MAX). Detailed summary statistics of all traits are shown in Table 1. The pedigree consisted of 1928 animal–sire–dam entries.

### 2.3. Genotypic Data

All pigs were genotyped using the PorcineSNP60 BeadChip (Illumina). The genotyping platform of Infinium II Multi-Sample Assay was used. SNP chips were scanned using iScan and analyzed using Illumina GenomeStudio (Illumina, Inc. 9885 Towne Centre Drive, San Diego, CA 92121, USA). To assess the technical reliability of the genotyping panel, two or more randomly selected DNA samples were genotyped, and over 99.9% identity was obtained. To update the SNP positions, we reordered the SNPs according to the newest version of the pig genome, *Sus scrofa* 11.1.

### 2.4. Quality Filtering

Quality control was performed using PLINK v1.90 [19]. The filtering criteria were (1) call rate higher than 0.98, (2) minor allele frequency (MAF) higher than 0.05, and (3) SNPs were filtered to exclude loci assigned to unmapped contigs and to sex chromosomes. After quality control, a total of 977 pigs and 31,144 SNPs were retained for the subsequent study.

### 2.5. Statistical Models

In this study, the heritability was estimated using the classic animal model as follows:

y=Xb+Za+e

where *y* is the vector of the phenotypic value of the trait under study; *b* is a vector of fixed effects, i.e., sex; *a* is a vector of additive genetic effect vector following N(0, A$\sigma_{g}^{2}$), where *A* is the numerator relationship matrix and $\sigma_{g}^{2}$ is the additive genetic variance; e is a vector of residuals following N(0, I$\sigma_{e}^{2}$), where I is an identity matrix and $\sigma_{e}^{2}$ is the residual variance; and *X* and *Z* are the incidence matrices relating phenotypes to fixed effects and random genetic effects, respectively.

In this study, teat number relevant traits were divided into two categories of counted and binary traits, so two kinds of mixed models were utilized for analysis.

For counted traits (TTN, LTN, RTN, MAX an ALR), the following model was applied:

y=μ+Xβ+Sα+u+e

For the binary trait (SLR), the following model was employed:

logit(y)=μ+Xβ+Sα+u

where *y* is the vector of the phenotypic value of the trait under study; *µ* is overall population mean; *β* is the vector of fixed effects: sex; *α* is the regression coefficient of substituting allele of the candidate SNP to be tested; *u* is the vector of polygenic effect; *X* is the incidence matrix regarding to observations to fixed effects; *S* is the incidence matrix relating observations to SNP effects with elements coded as 0, 1, 2 for homozygote of the reference allele, heterozygote, and homozygote of the alternative allele; *u* is the vector of random polygenic effects; and *e* is the vector of random residual error. The random effects *u* were assumed to be normally distributed with zero means and the following covariance structure $u\sim N\left(0,\;G\sigma_{u}^{2}\right)$, where *G* is the genomic relationship matrix (GRM) and $\sigma_{u}^{2}$ is the additive genetic variance, $e\sim N\left(0,\;I\sigma_{e}^{2}\right)$, where $\sigma_{e}^{2}$ is the residual variance.

Associations between counted or binary traits under study and the SNPs were analyzed one SNP at a time using GCTA software [20] and GenABEL package in R environment [21], respectively. Threshold p-values for suggestive and Bonferroni-adjusted genome-wide significance were set to −log10(p-value) = 4.49 (1/31,144 independent tests) and −log10(*p*-value) = 5.79 (0.05/31,144 independent tests), respectively.

### 2.6. Annotation of Candidate Genes

The porcine genome assembly 11.1 (http://ftp.ensembl.org/pub/release-103/fasta/sus_scrofa/dna/Sus_scrofa.Sscrofa11.1.dna.toplevel.fa.gz, accessed on 4 March 2021) and National Center for Biotechnology Information (NCBI) Genome (http://www.ncbi.nlm.nih.gov/genome/?term=pig, accessed on 4 March 2021) were retrieved to characterize candidate genes in targeted regions. The search to for positional candidate genes was extended 50 kb up- and downstream from the significant SNPs.

## 3. Results

### 3.1. Basic Description Statistics of Each Trait

The descriptive statistics of teat number relevant traits in Duroc population are shown in Table 1. The average numbers of LTN, RTN, TTN, and MAX were 6.55 ± 0.67, 6.61 ± 0.68, 13.17 ± 1.12, and 6.83 ± 0.63, respectively. The range of TTN was from 10 to 16. The pedigree-based heritabilities and genomic heritabilities of these seven traits were showed in Table 1 as well. As seen in Table 1, heritabilities of LTN, RTN, TTN, and MAX were 0.178, 0.228, 0.321, and 0.255, respectively, and all were larger than their corresponding genomic heritabilities. Heritabilities of ALR and SLR were low heritable, but pedigree-based heritabilities were smaller than their corresponding genomic heritabilities. For LR, both pedigree-based heritability and genomic heritability were nil.

### 3.2. Genome-Wide Association Study and Gene Annotation

**Figure 1:**
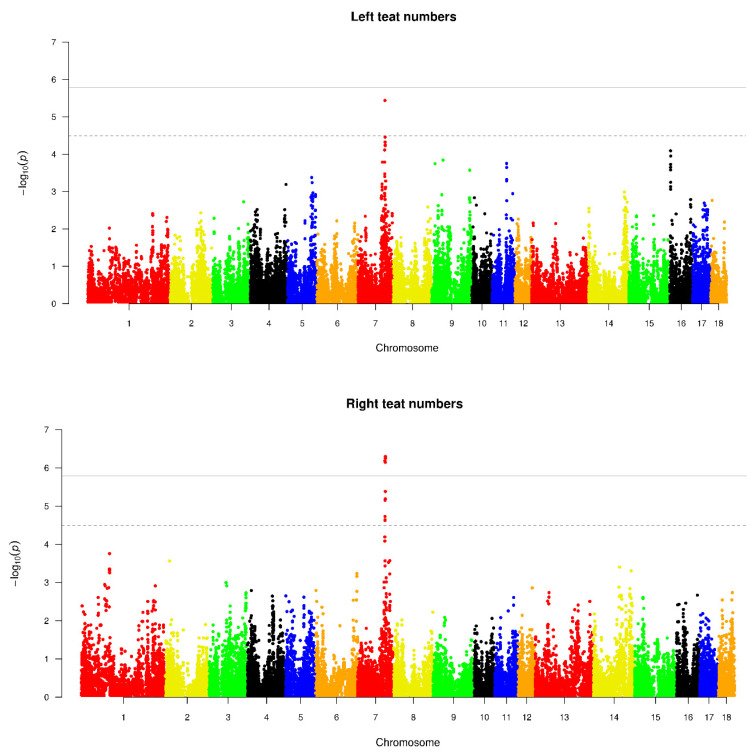

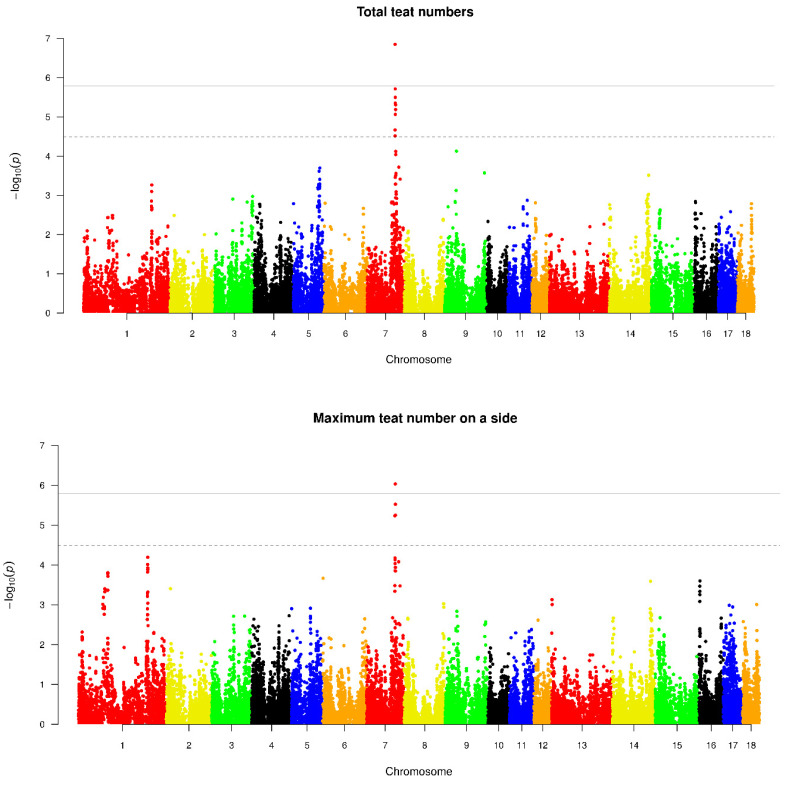

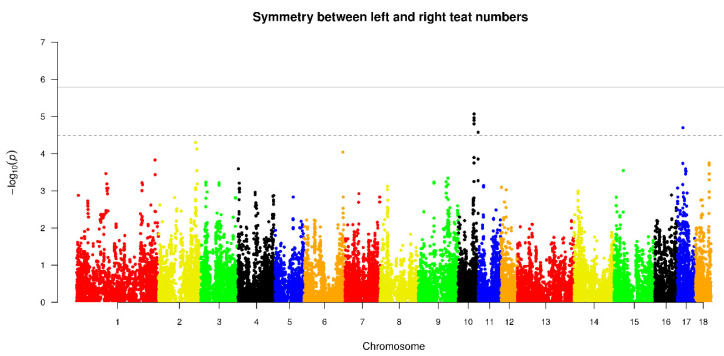
Manhattan plots representing genome-wide associations with five traits in the Duroc pig population. SNP −log10 (*p*-values) are shown across the 18 autosomal chromosomes. Black horizontal lines denote 0.05 genome-wide threshold (−log10(0.05/31,144) = 5.79), and dotted horizontal lines denote suggestive significant threshold (−log10(1/31,144) = 4.49).

Out of seven traits under study, there were significant SNPs detected only in five traits: TTN, RTN, LTN, MAX, and SLR. Figure 1 shows the Manhattan plots for these five traits. The corresponding QQ plots of the observed p-values against the expected p-values are exhibited in Figure S1. Table 2 displays the significant SNPs identified in these five traits and their annotated candidate genes. As seen in Figure 1 and Table 2, a total number of 13, 11, 1, 4, and 9 SNPs were found to be significantly associated with TTN, RTN, LTN, MAX, and SLR, respectively.

Totally, four and 22 SNPs surpassed genome-wide significance and suggestive significance levels, respectively (Table 2). Moreover, twelve SNPs were located in genic regions. Out of them, thirteen identified SNPs associated with TTN were concentrated on the genomic region between 96.10 and 98.09 Mb on *Sus scrofa* chromosome (SSC) 7 (Figure 1); significant SNPs associated with RTN, LTN, and MAX were located in this region as well. The most significant SNP (MARC0038565) in LTN, MAX, and TTN was associated with RTN, LTN, MAX, and TTN. Two annotated genes (*VRTN* and *SYNDIG*1*L*) resided in a span distance of 50 kb around the SNP (Table 2). Three significant SNPs (H3GA0035527, ASGA0035527, and INRA0027601) were shown to be associated with TTN, RTN and MAX traits. These three SNPs were lying in genomic regions harboring six candidate genes (*YLPM*1, *PROX*2, *DLST*, *RPS6KL*1, *DPF*3, and *ZFYVE*1). Seven significant SNPs (MARC0033479, DRGA0008025, ALGA0043926, MARC0048752, M1GA0010654, ALGA0043962, and INRA0027603) were simultaneously detected in TTN and RTN. Eight candidate genes (*NUMB*, *HEATR*4, *RIOX1*, *AREL*1, *FCF*1, *YLPM*1, *DNAL*1, and *MIDEAS*) were located in the genomic regions containing these seven SNPs. Other two SNPs (M1GA0010637 and ALGA0043904) were identified only in TTN and were located in *DPF3* gene region. Nine significant SNPs were shown to be significantly associated with SLR. Eight of them were located on SSC 10 and one on SSC 17. Two genes were annotated as *NRP1* and *ITBG1*. Among candidate genes annotated, eight genes have documented association with the teat number relevant traits. They were *VRTN*, *SYNDIG*1*L*, *YLPM*1, *PROX*2, *DPF*3, *NUMB*, *AREL1*, and *FCF1*.

## 4. Discussion

The teat number-relevant traits play an important role in rearing piglets and are easy to measure in both males and females. Although the teat number-relevant traits in pigs are characterized as a discontinuous counting variable. However, the observed distribution of the NT in our data was approximately followed the Gaussian distribution. It was showed that Gaussian distribution would be better fitted to this trait compared to the Poisson distribution [22,23].

In the current study, the estimated pedigree-based heritabilities of LTN, RTN, TTN, and MAX were larger than those of genomic-based heritabilities. This result is consistent with that of Gary A. Rohrer et al. [8]. Total teat numbers had the highest heritability of 0.49, while LTN and RTN are 0.38 and 0.30, respectively [8]. Lopes M S. et al. based on Landrace breed showed that TTN has a pedigree-based heritability of 0.37 [18], which is higher than that of this study. The heritabilities of pig teat numbers is moderately heritable, with an estimated value between 0.2 and 0.47 [5,24]. The heritability of LTN is higher than RTN, respectively, 0.20 and 0.18 reported by Borchers et al. [25]. In the current study, the number of pig teats had medium heritability. This indicates that the number of pig teat is less affected by environmental factors and is mainly affected by genetic factors. In addition, the pedigree-based heritabilities of ALR and SLR were smaller than those of genomic heritabilities. For LR, both pedigree-based heritability and genomic heritability were nil, as the additive genetic variance estimated was 1×10^−7^. The same result was reported by Gary A. et al. [8]., which was consistent with ours. Therefore, there is no need for further GWAS analysis for LR. This indica ted that LR and ALR were controlled by non-genetic factors.

In the present study, 13, 11, 1, and 4 SNPs were found to be associated with TTN, RTN, LTN, and MAX, respectively. These SNPs were located in one genomic region between 96.10 and 98.09 Mb on SSC 7. This genomic region is almost overlapped with a QTL hotspot on SSC 7 between 96.1 and 98.2 Mb (Sscrofa 11.1) that was also identified to be associated with TTN in the Canadian Duroc pig population by Zhuang et al. [9]. However, Zhuang et al. did not identify any significant SNPs around this region in American Duroc pig population. In this genomic region, Zhuang et al. found 37 significant SNPs identified in the Canadian Duroc population. Out of them, 10 SNPs were same with those in our study [9]. In Zhuang’s study, only the total number of teats was analyzed in two lines of Duroc pigs using multiply GWAS methods. The most important SNP (rs692640845) identified by Zhuang et al. did not show significant effect in our study. To the best of our knowledge, SLR was first investigated by the GWAS analysis in our study, although the other five traits (RTN, LTN, MAX, LR, and ALR) have been analyzed in the previous GWAS studies [8,26], significant SNPs identified are almost different.

It should be noted that one SNP (MARC0038565, chr7:97652632) was found to be simultaneous associated with RTN, LTN, MAX, and TTN in the present study. This SNP was also identified in Canadian Duroc pigs [9] and Large White pigs [26]. *VRTN* and *SYNDIG*1*L* resided in upstream about 28 kb and downstream 5 kb of the top SNP (MARC0038565), respectively. This was further confirmed that *VRTN* can be proposed as the most promising candidate gene; however, we still did not identify the genetic variants in this genic region related to number of teat relevant traits. It had been documented that *VRTN* had a major effect on the development of thoracic vertebrae (ribs) in pigs as well [27,28,29,30,31,32,33]. This suggested that vertebra and teat number were controlled by the common gene.

In this study, 18 genes were annotated genomic regions around the significant SNPs. Out of them, eight genes including *VRTN* and *SYNDIG*1*L* had shown to be associated with the teat number relevant traits. The most significant SNP identified in RTN was located on the *PROX2* genic region. Coupling with *PROX2*, *SYNDIG1L*, and *YLMP1* have been reported as the key candidate genes for teat number traits [2,34]. A study based on Duroc breed found the most significant SNP effect is in or near *FCF1* and *AREL1* genes associated with teat number [11]. There were two significant SNPs within genic regions of *NUMB* and *FCF1* associated with RTN and TTN. *NUMB* plays an important role in regulation on mammary development [35]. Lin et al. found that the downregulation of *DPF3* plays an indispensable function in the progression of breast cancer [36]. Furthermore, four significant SNPs related to SLR were located in the *NRP1* genic region. *NRP1* acts as a Neutrophil elastase (NE) receptor mediating uptake and PR1 cross-presentation in breast cancer cells [37]. The current findings, although in need of extensive validation, are expected to promote our understanding of the underlying regulatory mechanism of teat number traits in pigs.

Duroc sires are commonly utilized as a terminal sire in three-way crossbreeding programs. The breeding program of the Duroc pig has emphasized growth and efficiency traits rather than reproduction, therefore teat numbers and related traits have not been selected for in Duroc pig population in which there are theoretically ample of genetic variants on teat numbers. Zhuang et al. reported that significant SNPs identified in two different lines of Duroc pigs were totally different [9]. Moscatelli et al. identified one SNP in Large White pigs as being the same as in Duroc population [26]. These findings suggest that the genetic architecture of teat number traits is complex and genetic variants have population specific effects.

## 5. Conclusions

We carried out genome-wide association studies on teat number-relevant traits in a Duroc population. In total, there were nine and 22 SNPs exceeding genome-wide significance and suggestive significance levels, respectively. The identified SNPs are concentrated on SSC 7 and 10. Eighteen genes were annotated near them. Out of them, two new candidate genes were found: one is *DPF3* on SSC 7 for TTN and another is *NRP1* gene on SSC 10 for SLR. Our findings may provide the information resource to further understanding of the genetic mechanism of teat number relevant traits, and a reference to optimize breeding program for improvement of reproductive performances in pigs.

## Figures and Tables

**Table 1:** Summary statistics and Genomic heritablities of teat number relevant traits in Duroc pig population.

Traits	Mean ± sd	Rang	Pedigree-Based Heritability (SE)	Genomic-Based Heritability (SE)
LTN	6.55 ± 0.67	5–9	0.178 (0.060)	0.148 (0.044)
RTN	6.61 ± 0.68	5–8	0.228 (0.067)	0.177 (0.045)
TTN	13.17 ± 1.12	10–16	0.321 (0.077)	0.289 (0.053)
MAX	6.83 ± 0.63	5–9	0.255 (0.072)	0.220 (0.051)
LR	0.062 ± 0.76	−2–2	0.000 (0.028)	0.000 (0.024)
ALR	0.49 ± 0.59	0–2	0.017 (0.032)	0.054 (0.034)
SLR	-	-	0.010 (0.030)	0.032 (0.028)

sd, standard deviation; LTN, the left number of teats; RTN, the right number of teats; TTN, the total number of teats; MAX, maximum teat number on a side; LR, difference between RTN and LTN; ALR, the absolute value of LR; SLR, the presence of symmetry between left and right teat numbers.

**Table 2:** Significant SNPs associated with teat number relevant traits and their annotated candidate genes.

Trait	Chromosome	SNP	Position (bp)	*p* Value	Gene
**RTN**	7	H3GA0022664	98066911	5.03 × 10^−7^	*YLPM1, PROX2(within), DLST, RPS6KL1*
	7	ASGA0035527	98089286	5.66 × 10^−7^	*YLPM1, PROX2, DLST, RPS6KL1*
	7	INRA0027601	96278617	6.55 × 10^−7^	*DPF3, ZFYVE1*
	7	MARC0038565	97652632	7.17 × 10^−7^	*VRTN, SYNDIG1L*
	7	MARC0048752	97946666	4.06 × 10^−6^	*AREL1, FCF1(within), YLPM1*
	7	M1GA0010654	97954258	6.52 × 10^−6^	*AREL1, FCF1(within), YLPM1*
	7	ALGA0043962	97973860	6.52 × 10^−6^	*AREL1, FCF1, YLPM1*
	7	INRA0027603	97048513	7.06 ×10^−6^	*DNAL1(within), MIDEAS*
	7	MARC0033479	96786713	2.39 × 10^−5^	*NUMB(within), HEATR4, RIOX1*
	7	DRGA0008025	96731837	1.87 × 10^−5^	*NUMB(within)*
	7	ALGA0043926	96806775	2.28 × 10^−5^	*NUMB, HEATR4, RIOX1*
**LTN**	7	MARC0038565	97652632	3.66 × 10^−6^	*VRTN, SYNDIG1L*
**MAX**	7	MARC0038565	97652632	9.28 × 10^−7^	*VRTN, SYNDIG1L*
	7	H3GA0022664	98066911	5.67 × 10^−6^	*YLPM1, PROX2(within), DLST, RPS6KL1*
	7	ASGA0035527	98089286	2.99 × 10^−6^	*YLPM1, PROX2, DLST, RPS6KL1*
	7	INRA0027601	96278617	5.86 × 10^−6^	*DPF3, ZFYVE1*
**TTN**	7	MARC0038565	97652632	8.67 × 10^−9^	*VRTN, SYNDIG1L*
	7	H3GA0022664	98066911	6.40 × 10^−8^	*YLPM1, PROX2(within), DLST, RPS6KL1*
	7	ASGA0035527	98089286	5.22 × 10^−8^	*YLPM1, PROX2, DLST, RPS6KL1*
	7	INRA0027601	96278617	1.41 × 10^−7^	*DPF3, ZFYVE1*
	7	MARC0033479	96786713	4.44 × 10^−6^	*NUMB(within), HEATR4, RIOX1*
	7	DRGA0008025	96731837	3.19 × 10^−6^	*NUMB(within)*
	7	ALGA0043926	96806775	1.93 × 10^−6^	*NUMB, HEATR4, RIOX1*
	7	MARC0048752	97946666	4.93 × 10^−6^	*AREL1, FCF1(within), YLPM1*
	7	M1GA0010654	97954258	6.52 × 10^−6^	*AREL1, FCF1(within), YLPM1*
	7	ALGA0043962	97973860	6.52 × 10^−6^	*AREL1, FCF1, YLPM1*
	7	INRA0027603	97048513	8.66 × 10^−6^	*DNAL1(within), MIDEAS*
	7	M1GA0010637	96101509	2.16 × 10^−5^	*DPF3(within)*
	7	ALGA0043904	96128654	3.06 × 10^−5^	*DPF3(within)*
**SLR**	10	MARC0112026	56303425	8.58 × 10^−6^	*NRP1(within)*
	10	ALGA0102459	56421545	8.58 × 10^−6^	*NRP1(within)*
	10	ASGA0090893	56211944	1.09 × 10^−5^	*ITBG1*
	10	MARC0010633	56282915	1.27 × 10^−5^	*NRP1(within)*
	10	MARC0052641	56340616	1.27 × 10^−5^	*NRP1(within)*
	17	H3GA0048042	19474175	2.01 × 10^−5^	*-*
	10	ASGA0089631	56237188	1.59 × 10^−5^	*NRP1*
	10	ASGA0098692	56259450	1.59 × 10^−5^	*NRP1*
	10	MARC0068931	55591111	2.67 × 10^−5^	*-*

LTN, the left number of teats; RTN, the right number of teats; TTN, the total number of teats; MAX, maximum teat number on a side; SLR, the presence of symmetry between left and right teat numbers.

## Data Availability

The data presented in this study are available on request from the corresponding author.

## Supplementary Materials

The following are available online at https://www.mdpi.com/2076-2615/11/3/806/s1, Figure S1: The Q-Q plots obtained from genome-wide association studies for teat number.
