# Supplementary material for: Revealing New Candidate Genes for Teat Number Relevant Traits in Duroc Pigs Using Genome-Wide Association Studies
Source: Animals (Basel). 2021 Mar 13;11(3):806. doi: 10.3390/ani11030806 (PMC7998181; doi:10.3390/ani11030806)
Supplement: Supplementary file 1 [file animals-11-00806-s001.pdf]

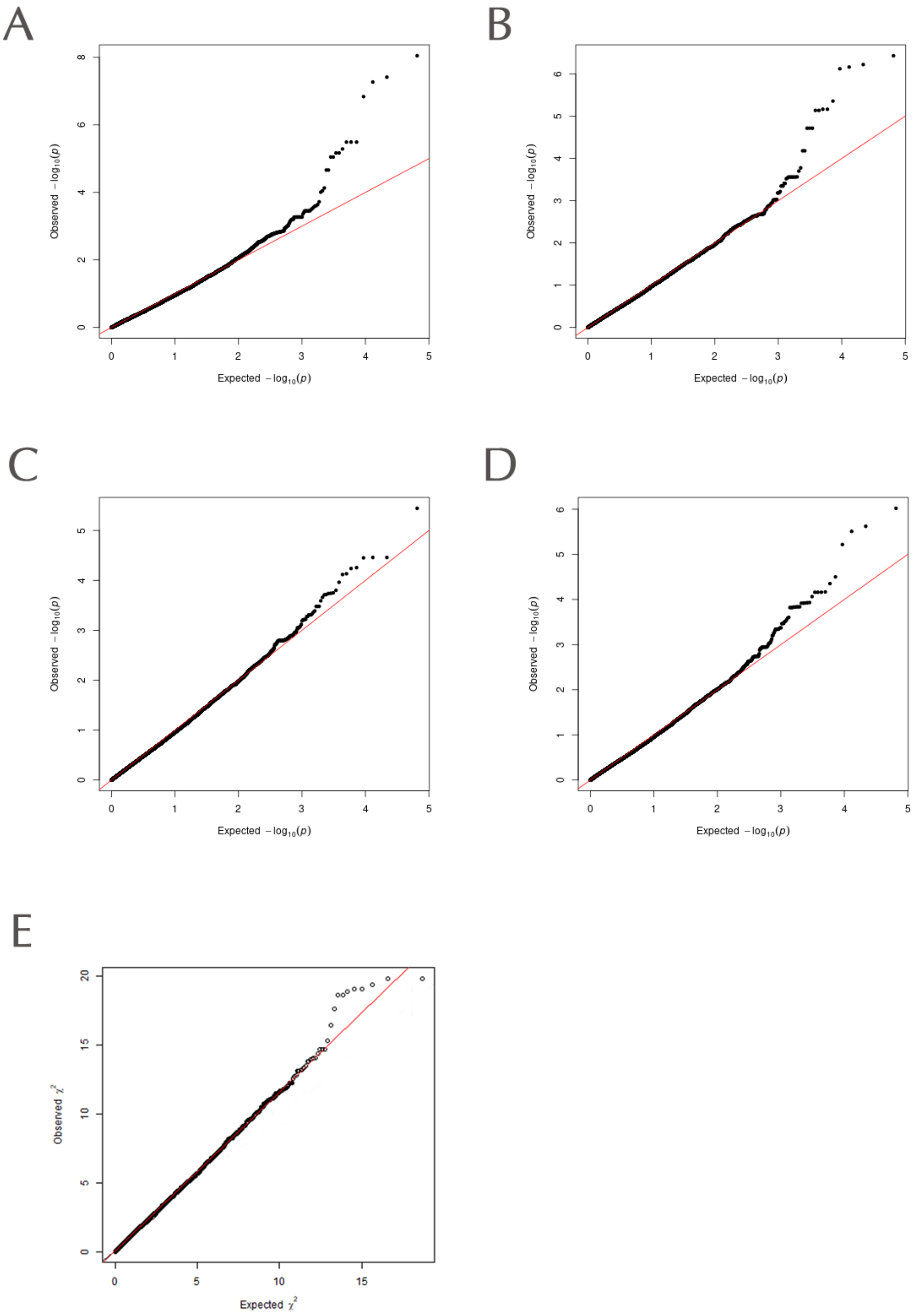

Figure S1. A, B, C, D and **E** were the Q-Q plot of total teat numbers, right teat numbers, left teat numbers, maximum number of teats on a side and symmetry between left and right teat numbers. The Q-Q plots of A, B, C and D show the observed -log10 transformed *p*-values (y-axis) and the expected -log10 transformed *p*-values (x-axis). The Q-Q plots of E show the observed χ^2 transformed *p*-values (y-axis) and the expected χ^2 transformed *p*-values (x-axis)
